# Comprehensive meta-analysis of surgical procedure for congenital diaphragmatic hernia: thoracoscopic versus open repair

**DOI:** 10.1007/s00383-024-05760-7

**Published:** 2024-07-09

**Authors:** Soichi Shibuya, Irene Paraboschi, Stefano Giuliani, Takafumi Tsukui, Andreea Matei, Maricarmen Olivos, Mikihiro Inoue, Simon A. Clarke, Atsuyuki Yamataka, Augusto Zani, Simon Eaton, Paolo De Coppi

**Affiliations:** 1 Stem Cell and Regenerative Medicine Section, Developmental Biology and Cancer Research & Teaching Department, Zayed Centre for Research Into Rare Disease in Children, Great Ormond Street Institute of Child Health, University College London, 30 Guilford Street, London, WC1N 1E UK; 2 Department of Pediatric General and Urogenital Surgery, Juntendo University School of Medicine, 3-1-3 Hongo, Bunkyo City, Tokyo, 113-8431 Japan; 3 Department of Biomedical and Clinical Science, University of Milano, Milan, Italy; 4 Department of Specialist Neonatal and Paediatric Surgery, Great Ormond Street Hospital for Children, NHS Foundation Trust, London, UK; 5 Division of General and Thoracic Surgery, The Hospital for Sick Children, Toronto, ON Canada; 6 Chelsea and Westminster NHS Foundation Trust, London, UK; 7 Department of Pediatric Surgery, Fujita Health University, Aichi, Japan; 8 Department of Surgery, University of Toronto, Toronto, ON Canada

**Keywords:** Congenital diaphragmatic hernia, Meta-analysis, Minimally invasive surgery, Thoracoscopy, Neonatal surgery, Bowel obstruction

## Abstract

**Purpose:**

Previous studies have shown a higher recurrence rate and longer operative times for thoracoscopic repair (TR) of congenital diaphragmatic hernia (CDH) compared to open repair (OR). An updated meta-analysis was conducted to re-evaluate the surgical outcomes of TR.

**Methods:**

A comprehensive literature search comparing TR and OR in neonates was performed in accordance with the PRISMA statement (PROSPERO: CRD42020166588).

**Results:**

Fourteen studies were selected for quantitative analysis, including a total of 709 patients (TR: 308 cases, OR: 401 cases). The recurrence rate was higher [Odds ratio: 4.03, 95% CI (2.21, 7.36), *p* < 0.001] and operative times (minutes) were longer [Mean Difference (MD): 43.96, 95% CI (24.70, 63.22), *p* < 0.001] for TR compared to OR. A significant reduction in the occurrence of postoperative bowel obstruction was observed in TR (5.0%) compared to OR (14.8%) [Odds ratio: 0.42, 95% CI (0.20, 0.89), *p* = 0.02].

**Conclusions:**

TR remains associated with higher recurrence rates and longer operative times. However, the reduced risk of postoperative bowel obstruction suggests potential long-term benefits. This study emphasizes the importance of meticulous patient selection for TR to mitigate detrimental effects on patients with severe disease.

**Supplementary Information:**

The online version contains supplementary material available at 10.1007/s00383-024-05760-7.

## Introduction

Congenital diaphragmatic hernia (CDH) remains one of the most challenging congenital malformations, with a mortality rate ranging from 10 to 35% [1–3]. Long-term morbidity in survivors is common and affects various organs, necessitating a multidisciplinary approach in follow-up care to improve the patients' quality of life [4–7]. Over the last two decades, minimally invasive surgery (MIS) has emerged as an alternative to conventional open repair (OR) for CDH [8–11]. While laparoscopic repair was also initially performed, thoracoscopic repair (TR), which has been first reported in neonates by Liem at el., is now considered the standard MIS technique for CDH repair [11–17]. Despite its potential benefits, MIS has not yet become the standard procedure and continues to be a subject of controversy [18–21]. This is highlighted by previous meta-analyses that have shown drawbacks of MIS, such as longer operative times and higher recurrence rates compared to OR [22–25]. Several studies have also suggested that TR is associated with an increased risk of intraoperative hypercapnia and acidosis [26–28], prompting further research into optimal ventilation strategies during surgery [29–31]. Considering these findings, recent guidelines for the treatment of CDH have excluded TR from the recommended standard surgical procedures [6, 32, 33]. Nonetheless, TR continues to be performed in many institutions, underpinned by the belief that initial challenges can be addressed through increased experience and refined management [29, 34, 35]. This study aims to re-evaluate the efficacy and suitability of TR for CDH by conducting an updated meta-analysis that incorporates original data from experienced centers.

## Materials and methods

### Study background

The study adhered to the Preferred Reporting Items for Systematic Reviews and Meta-Analyses (PRISMA) statement and followed the guidelines outlined in the Meta-analysis Of Observational Studies in Epidemiology (MOOSE) checklist [36–38]. Before commencing the research, a pre-defined protocol was registered in PROSPERO (CRD42020166588). The study focused on CDH patients who underwent surgical treatment during the neonatal period. To maintain consistency in the subject, the study excluded cases of late-onset diaphragmatic hernia (i.e., repaired more than 30 days after birth), as well as minimally invasive surgeries other than TR (e.g., laparoscopy, robotic-assisted surgery), Morgagni hernia, and cases post fetal therapy.

### Search strategy

A thorough literature search was initially conducted in August 2020 by two clinical investigators (SS and IP), followed by an additional search in January 2024 to include the most recent publications. The search spanned electronic databases, including Medline, Embase, Web of Science, and Cochrane Central Register of Controlled Trials (CENTRAL), using a combination of search terms: (“diaphragmatic hernia”) AND (“thoracoscop*” OR “minimally invasive”). The screening process involved the assessment of titles and abstracts, with subsequent evaluation of full-text articles in accordance with the predefined inclusion and exclusion criteria. Discrepancies in the selection were resolved through consensus among the authors (SS, IP, SE, SG, and PDC). The Methodological Index for Non-Randomized Studies (MINORS) score was employed to assess the quality of the studies [39]. Conference abstracts and unpublished materials meeting the inclusion criteria were also included, provided that sufficient raw data were available from the authors.

### Method of data extraction and evaluation

Primary outcome measures, including the recurrence rate (without differentiating between early and late recurrences) and operative time, were selected according to the recognized disadvantages of TR. Subgroup analyses were conducted based on the year of surgery, defect size, and patch usage. Secondary outcomes included the duration of postoperative ventilation, total hospital stay, and the occurrence of postoperative bowel obstruction, which are regarded as benefits of TR. Sensitivity analyses were performed to account for inconsistencies across studies regarding the treatment of conversion cases. Corresponding authors of selected papers were contacted for data clarification as necessary. Means and standard deviations were estimated from median values and range or interquartile ranges using the methods outlined by Wan et al. [40].

### Statistical analysis

Categorical variables were compared using the Chi-square test. Given the diverse study designs, a random-effects model was chosen for the quantitative analysis. Effect estimates for continuous data were reported as weighted mean differences (WMD), while dichotomous variables were assessed as odds ratios. Heterogeneity was evaluated using the I2 statistic. Effect estimates were presented with 95% confidence intervals (CIs), with statistical significance set at *p* < 0.05. Funnel plots were used to assess publication bias. The statistical analyses were conducted using Review Manager (RevMan) version 5.4.1 (The Cochrane Collaboration, 2020).

## Results

### Study inclusion

**Fig. 1:**
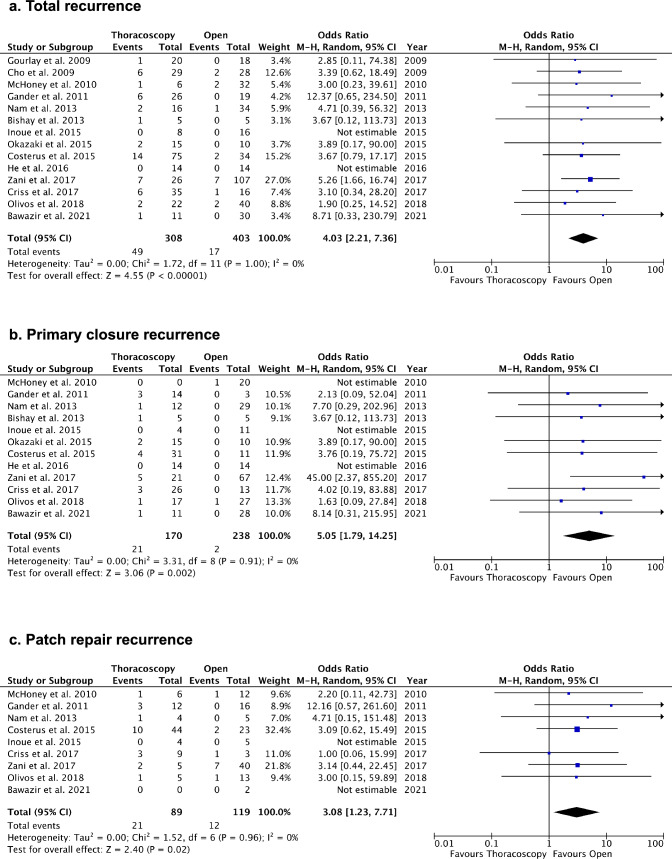
PRISMA flow chart

The study selection process is visually depicted in the PRISMA diagram (Fig. 1). Fourteen English-language studies were included in the quantitative synthesis, as detailed in Table 1 [16, 18, 20, 26–28, 30, 41–47]. One conference proceeding was incorporated due to the availability of complete data from the corresponding author [46]. In addition, although one study included non-neonatal data, it was deemed eligible, because the authors provided the raw data, allowing for data refinement [42]. A total of 709 patients were analyzed, with 308 cases in the TR group and 401 cases in the OR group. Among the TR cases, 52 (15.2%) were converted to OR. Some studies included these conversion cases, while others excluded them. These conversion cases (*n* = 30) were included in our primary meta-analysis. Additional sensitivity analyses that excluded conversion cases were conducted to ensure robustness and minimize potential confounding factors associated with conversions.

**Table 1:** Summary of the studies included in the meta-analysis

				Study Period (year)			Patients (n)			Conversion		Patch repair (n)		Follow-up period (months^a^)		
Reference	Year	Institutions	Design of Study	TR	OR	Additional data	Total	TR	OR	Included/ Excluded	n	TR	OR	TR	OR	MINORS score
Cho et al	**2009**	Portland, Oregon, USA	Comparative study with historical control	2004–2007	2001–2004	–	57	29	28	Included in TR	1	15	12	11.2 ± 1.9	8.1 ± 1.8	11
Gourlay et al	**2009**	Milwaukee, Wisconsin, USA	Comparative study with matched historical control	2004–2007	1999–2003	–	38	20	18	Excluded	1	4	8	14.5	37	10
McHoney et al	**2010**	London, United Kingdom	Comparative study	2007–2008	2003–2008	Obtained	38	6	32	Excluded	5	6	12	15 (1–40)	31 (5–84)	13
Gander et al	**2011**	Columbia, New York, USA	Comparative study	2006–2010	2006–2010	–	45	26	19	Excluded	9	12	16	14 (1–47)	14 (1–35)	12
Bishay et al	**2013**	London, United Kingdom	Randomized control trial	2009–2011	2009–2011	Obtained	10	5	5	-	0	0	0	14.5 (3–23)	13.3 (8–13.5)	21
Nam et al	**2013**	Seoul, South Korea	Comparative study	2008–2011	2008–2011	–	50	16	34	Included in TR	2	4	5	–	–	13
Inoue et al	**2015**	Mie, Japan	Comparative study with matched historical control	2010–2014	2000–2009	Obtained	24	8	16	-	0	4	5	42 (4–59)	94.5 (75–171)	12
Okazaki et al	**2015**	Tokyo, Japan	Comparative study	2007–2014	2002–2014	Obtained	25	15	10	Excluded	5	0	0	110 (91–157)	205 (152–229)	13
Costerus et al	**2016**	Rotterdam, Netherlands and Mannheim, Germany	Comparative study	2008–2012	2008–2012	–	109	75	34	Included in TR	15	44	23	12	12	17
He et al	**2016**	Guangzhou, China	Comparative study with historical control	2013–2014	2011–2013	–	28	14	14	Included in TR	1	0	0	12	12	11
Zani et al	**2017**	Toronto, Ontario, Canada	Comparative study	2004–2014	2004–2014	Obtained	133	26	107	Included in OR	6	6	12	96 (9–167)	92 (14–188)	15
Criss et al	**2018**	Ann Arbor, Michigan, USA	Comparative study	2006–2016	2006–2016	–	49	35	14	Excluded	2	9	3	19 (1–102)	48 (13–74)	15
Olivos et al	**2018**	Chelsea, United Kingdom	Comparative study	2005–2018	2005–2018	Obtained	62	22	40	Included in OR	3	4	10	26 (4–70)	21 (4–64)	10
Bawazir et al	**2021**	Makkah, Saudi Arabia	Comparative study	2011–2019	2011–2019	–	41	11	30	Included in OR	2	0	2	12	12	14

^a^Follow-up period is reported in either mean only or mean ± standard deviation or median (range)

*USA* United States of America, *TR* Thoracoscopic repair, *OR* Open repair

### Primary outcomes

**Fig. 2:**
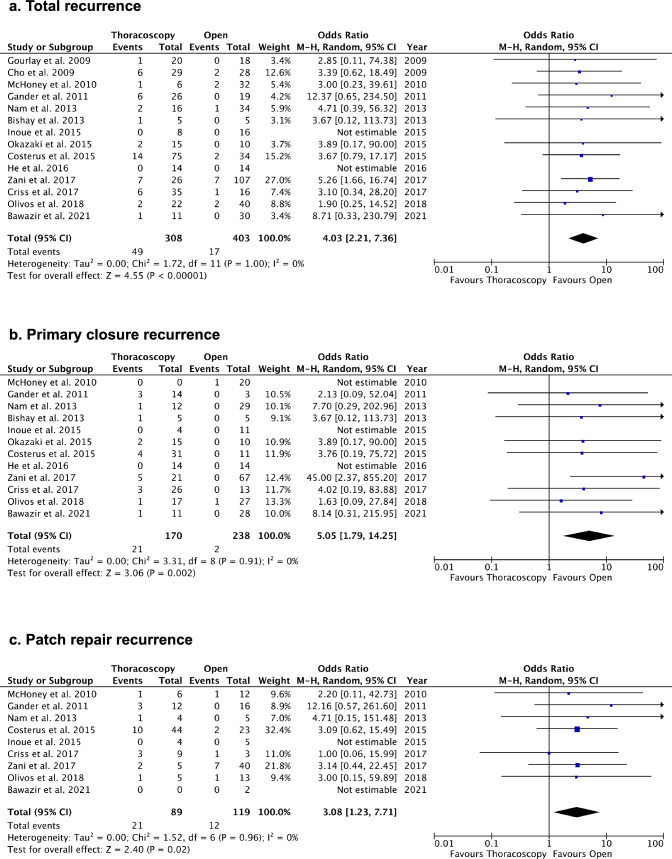
Comparisons of recurrence rate. **a** Total recurrence. **b** Primary closure recurrence. **c** Patch repair recurrence

**Fig. 3:**
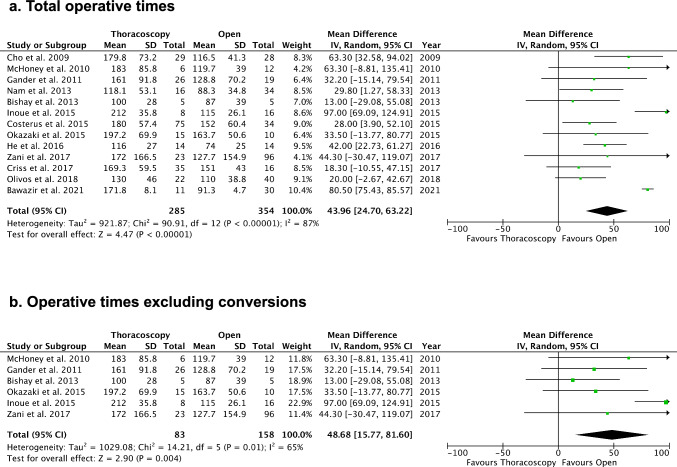
Comparisons of operative times. **a** Total operative times (minutes). **b** Operative times excluding conversions (minutes)

Most studies (85.7%) reported a higher recurrence rate for TR compared to OR (Fig. 2). The pooled recurrence rate for TR was 15.9% (49/308), while that for OR was 4.0% (17/401). The meta-analysis showed a significantly higher recurrence rate after TR [Odds ratio: 4.03, 95% CI (2.21, 7.36), p < 0.001] with very low heterogeneity (I^2^ = 0%). We additionally performed subgroup analyses to seek the correlation between patch usage and recurrence rate. The ratio of patch usage was similar between TR (35.1%; 108/308) and OR (34.7%; 139/401) [Odds ratio: 0.80, 95% CI (0.45, 1.39), *p* = 0.43], with moderate heterogeneity (I^2^ = 43%). Subgroup analyses for patch and primary repair indicated a higher recurrence rate for TR in both primary repair (12.4%) and patch repair (23.6%). Notably, recurrence rates were also comparatively higher after patch repair in both TR (23.6% vs. 13.2%) and OR (10.1% vs. 0.9%) (Suppl. 1a, Suppl. 1b), suggesting that patch usage is associated with higher recurrence rates irrespective of surgical procedure. All studies reported operative times, revealing a significantly longer operative time (minutes) for TR [MD: 43.96, 95% CI (24.70, 63.22), *p* < 0.001], with considerable heterogeneity (I^2^ = 87%). Sensitivity analysis excluding conversion cases yielded similar results (Fig. 3b).

### Secondary outcomes

**Fig. 4:**
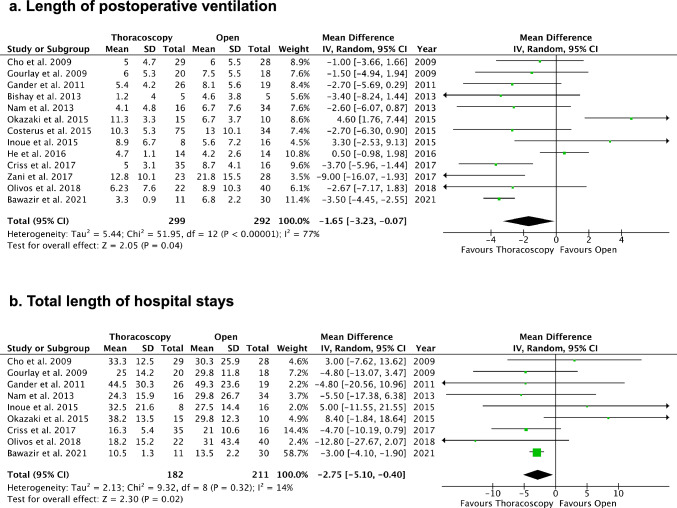
Comparisons of length of postoperative ventilation and total length of hospital stays. **a** Length of postoperative ventilation (days). **b** Total length of hospital stays (days)

**Fig. 5:**
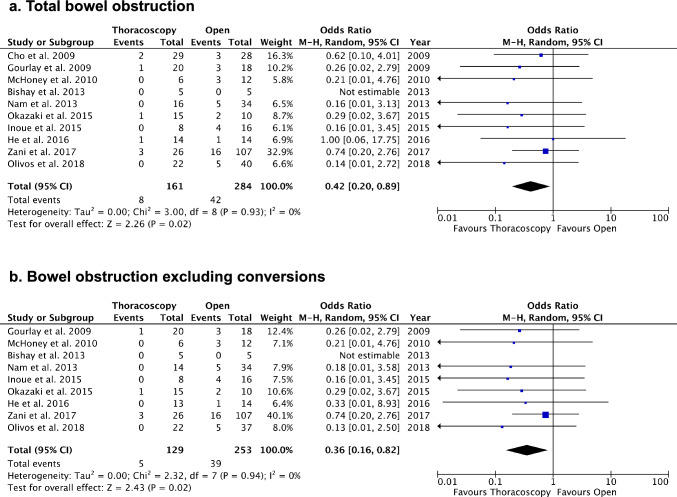
Comparisons of occurrence of bowel obstruction. **a** Total bowel obstruction. **b** Bowel obstruction excluding conversions

The meta-analysis showed a statistically shorter duration of postoperative ventilation (days) for TR [MD: −1.65, 95% CI (−3.23, −0.07), *p* < 0.05], with considerable heterogeneity (I2 = 77%) (Fig. 4a). Total length of hospital stay (days) was significantly shorter for TR [MD: −2.75, 95% CI (−5.10, −0.40), *p* < 0.05], with low heterogeneity (I^2^ = 14%) (Fig. 4b). In addition, the occurrence of postoperative bowel obstruction was lower after TR compared to OR (5.0% vs. 14.8%) [Odds ratio: 0.42, 95% CI (0.20, 0.89), p = 0.02], with very low heterogeneity (I^2^ = 0%) (Fig. 5a). Sensitivity analysis excluding conversion cases similarly indicated a reduced risk of postoperative bowel obstruction in TR [Odds ratio: 0.36, 95% CI (0.16, 0.82), p = 0.02, I^2^ = 0%] (Fig. 5b), thus excluding the impact of conversions on the results.

### Subgroup analyses

Subgroup analysis for cases performed after 2011 indicated a continued longer operative time for TR, although no significant difference in recurrence rate was observed (Suppl. 2a, Suppl. 2b). Another subgroup analysis focused on cases with small defects revealed no significant differences in operative times and recurrence rates (Suppl. 3a, Suppl. 3b).

### Risk of reporting *bias*

Funnel plots were generated for primary outcomes (recurrence and operative times) to assess the risk of reporting bias. The funnel plot for recurrence demonstrated a symmetric distribution of studies, while the plot for operative time exhibited some studies outside the 95% confidence interval lines, likely attributable to heterogeneity across studies rather than reporting bias (Suppl. 4a, Suppl. 4b).

## Discussion

Due to the severity and rarity of CDH, there is considerable controversy regarding the efficacy of MIS for its treatment. As of 2023, four meta-analyses have been published on this subject. The most recent one, released in 2016, included studies conducted up until 2013 [22–25]. These earlier meta-analyses had several limitations, such as the inclusion of both laparoscopic repairs and cases of late-onset CDH, which may have confounded their results. More recently, another systematic review was published that compared MIS to OR [48]. This study synthesized pooled data and reported findings similar to those of our meta-analysis, with the exception of bowel obstruction rates. It included a total of 32 publications; however, there was a noted inconsistency in the definition of MIS, which encompassed both laparotomy and thoracotomy, as well as an overlap in study cohorts. These issues highlight the challenges in conducting a comprehensive systematic review for this rare disease. In our current study, we implemented stricter selection criteria, which resulted in the exclusion of certain studies previously considered. We also performed sensitivity analyses to address the impact of remaining confounding factors, such as conversion cases. These analyses generally corroborated our primary results. Moreover, we took the proactive step of contacting the authors of the selected studies, successfully obtaining raw data from six out of the fourteen studies [16, 26, 27, 30, 42, 46]. We believe these steps substantially improved the validity of our meta-analysis, offering the most up-to-date and trustworthy evidence on the topic.

The primary objective of this study was to assess whether the surgical outcomes of TR have improved over the last two decades. The multinational CDH Study Group (CDHSG) reported a higher risk of recurrence during the initial hospitalization (early recurrence) after MIS, regardless of the defect size, emphasizing the need for further analysis focused on defect sizes to evaluate the impact of technical advancements in TR for reducing recurrence rates [49]. While there is a report demonstrating a declining trend in the early recurrence rate of TR [50], our study demonstrates a persistently higher recurrence rate in TR compared to OR [48, 51, 52]. To assess the effect of learning curve on the recurrence rate, we performed a subgroup analysis focusing on cases performed after 2011, which revealed no statistically significant difference in recurrence rates between TR (5.7%) and OR (1.5%). Although the statistical power is low due to the small number of cases included in the subgroup analysis (TR: 35, OR: 65), considering the notably higher recurrence rates of TR (15.9%) and OR (4.0%) in the overall result, there is a sign of convergence of the recurrence rates between TR and OR, suggesting the effect of learning curve on the high recurrence rate in the early period. The higher recurrence rate after TR can possibly be attributed to technical challenges in securely suturing the diaphragmatic defect, especially in cases with larger defects or less adhesion in the abdominal organs after TR, which makes migration of bowels easier [49]. In terms of patch usage, known as a risk factor for recurrence in OR, our subgroup analyses indicated variations in the preference for patch usage among institutions. Some institutions tend to use patches liberally in TR, while others exhibit hesitancy, as evidenced by the survey conducted by the International Pediatric Endosurgery Group (IPEG), which indicated that 31% of surgeons avoid using patches in TR and opt for conversion to OR if patch application is necessary [17]. Some advocate that liberal use of patch, even for medium-sized defects, is effective to reduce suture tension and increase the capacity of the abdominal cavity [45, 50, 53–57]. Although patch application was previously considered difficult in TR during the earlier period, improvements in surgical techniques have now made patch repair feasible even with a thoracoscopic approach [55]. Our subgroup analyses revealed a higher recurrence rate in TR compared to OR, regardless of patch usage, thus failing to demonstrate a beneficial effect of patch usage in preventing recurrence. However, the meta-analysis was unable to distinguish whether patches were used to close defects that were too large for primary closure or as reinforcement for relatively small defects. This ambiguity makes it difficult to evaluate the impact of liberal patch usage. Further research, incorporating details about the size of the defect and the shape of the patch, is required to determine whether a more flexible approach to patch usage can effectively reduce recurrence rates in TR [45, 55].

Regarding operative time, our study confirms longer operative times for TR compared to OR, in line with the previous meta-analyses. The primary cause of prolonged times is likely the technical challenges associated with thoracoscopic suturing in confined spaces. Although it has been reported that operative times may decrease as surgeons gain experience [55], the rarity of CDH makes it challenging to accumulate substantial experience with TR unless it is performed in centralized, high-volume centers. While lung hypoplasia and the duration of invasive ventilation are the primary factors affecting the long-term respiratory morbidity in patients, prolonged operative times also raise concerns. Extended surgical durations pose risks of hypercapnia and acidosis, which can be detrimental if the patient is exposed for extended periods [18, 26, 42, 58]. Although some studies have evaluated respiratory status during surgery, a quantitative synthesis of hypercapnia and acidosis was unfeasible in this meta-analysis due to significant inconsistencies in assessment methods across the studies. One randomized controlled trial (RCT) demonstrated significantly higher PaCO2 and lower pH levels during TR compared to OR [26]. However, a subsequent study by the same group showed that lowering insufflation pressure (from up to 10 mmHg in the RCT to 4 to 7 mmHg) significantly improved hypercapnia and acidosis [29]. In addition, intraoperative ventilatory strategies, including intrapulmonary percussive ventilation and high frequency oscillatory ventilation, have been suggested to alleviate hypercapnia, indicating that optimizing intraoperative respiratory management can help prevent severe acidosis during TR [30, 31]. ﻿A recent study comparing intraoperative blood gas parameters after ECMO between TR and OR found no significant differences, underscoring the safety of TR in such patients [59]. Nevertheless, concerns remain about the potential adverse effects of prolonged general anesthesia on neurological development. Although a recent RCT found no differences in neurodevelopmental outcomes between general and regional anesthesia for procedures lasting less than an hour, the safety of general anesthesia for longer durations, especially in small babies, has not yet been established [60, 61]. Therefore, it is crucial to minimize operative times and conduct thorough neurodevelopmental assessments for survivors to understand the long-term consequences.

Several recent studies, including one from the CDHSG, have reported significantly fewer occurrences of small bowel obstruction after MIS compared to OR, which is consistent with the findings of this meta-analysis [49, 62–64]. The incidence of bowel obstruction after OR has been reported to range from 10 to 25%, primarily associated with adhesions of the bowels at the repair site [4, 62–65]. In contrast, the incidence of bowel obstruction after TR has been reported to be less than 10% [18, 62–64, 66, 67]. Our pooled data align with these reports, demonstrating similar ratios of occurrences (14.8% vs. 5.0%). While early concerns suggested that non-anatomical bowel reduction in TR might increase the risk of volvulus related to malrotation, very few cases requiring subsequent Ladd's procedure have been reported thus far [18]. Notably, none of the studies included in this meta-analysis reported volvulus related to malrotation, implying that routine inspection for malrotation may be unnecessary. Considering that bowel obstruction may necessitate multiple hospitalizations and result in bowel loss due to additional surgeries, the reduction in the risk of bowel obstruction represents a long-term benefit of TR for survivors. A patient-led survey highlighted that a significant number of CDH survivors endure long-term feeding problems, significantly compromising their daily activities [7]. Although various factors, such as gastroesophageal reflux and neurodevelopmental delays, may contribute to feeding issues, the reduced bowel adhesion resulting from TR has the potential to ameliorate these long-term issues. Further studies are required to elucidate the positive effects of TR on the feeding problems of survivors.

We acknowledge several limitations in this study, the most prominent being the heterogeneity of the study designs across the included papers. Despite employing stringent selection criteria, it was challenging to eliminate the variability in patient cohorts due to the nature of the disease. Some studies exclusively selected mild cases or utilized matched historical controls to balance preoperative patient conditions, while others included all consecutive cases with or without selection criteria for TR. Consequently, the severity of the patients compared in our analysis was heterogeneous. To mitigate the effect of this heterogeneity, we intended to perform subgroup analyses based on the severity of the disease, but only two of the included studies reported the size of the defect, rendering such analyses unfeasible [50, 68]. The small number of cases identified as recently treated represents another limitation. Most papers include all patients without categorizing them by the period of operation, resulting in a significant number of cases treated during the learning curve period—some of which overlap with previously published meta-analyses—being included in our data. Furthermore, the number of cases handled by each center varies, suggesting that inconsistencies in surgeons’ experience may affect the data. These factors may have contributed to the longer operative times and higher recurrence rates persist in TR. Moreover, perioperative management of CDH is primarily based on institutional or individual surgeons' experiences, far from being standardized, and numerous factors may influence patient outcomes besides the surgical procedure itself, such as the indication for extracorporeal membrane oxygenation (ECMO), timing of surgery, and postoperative ventilation strategy. In addition, the follow-up period varied, potentially confounding the results for recurrence and bowel obstruction. Finally, the availability of RCTs and prospective studies was limited. Nevertheless, considering the complexity of conducting such studies for CDH, we believe that our meta-analysis represents the most appropriate methodological approach at present.

Despite technical adjustments over the last two decades, TR continues to result in a higher recurrence rate and longer operative times compared to OR. However, our subgroup analysis focusing on cases treated after 2011 suggested a trend toward decreasing recurrence rates in TR in more recent cases. Moreover, the risk of postoperative bowel obstruction is significantly lower after TR, which may offer long-term benefits to survivors. Given other potential benefits that are not easily objectively assessed, such as improved aesthetic outcomes and reduced pain, further trials of TR may be justified in experienced centers. Nevertheless, these positive results for TR should be interpreted with caution, as the study includes significant heterogeneity in patients, especially in terms of severity of lung hypoplasia and defect size. Considering the detrimental effects of recurrence and prolonged operative times on patients’ morbidity, careful selection of candidates is crucial to ensure operative safety, and further research is recommended to evaluate the effects of the learning curve and liberal patch usage.

## Supplementary Information

Below is the link to the electronic supplementary material.

Supplementary file 1: Suppl.1 Comparisons of recurrence rate according to patch usage. a) Open patch repair vs primary closure in open surgery. b) Thoracoscopic patch repair vs primary closure in thoracoscopic surgery

Supplementary file 2: Suppl.2 Subgroup analysis including only the cases later than 2011. a) Recurrence rate. b) Operative times (minutes)

Supplementary file 3: Suppl.3 Subgroup analysis including only the cases with a small defect. a) Recurrence rate. b) Operative times (minutes) *A small defect is defined as either of the following: 1) Type A or B defect according to The Congenital Diaphragmatic Hernia Study Group (Larry et al., 2013, J Pediatr Surg). 2) Maximum diameter of the defect is less than 5 cm

Supplementary file 4: Suppl.4 Funnel plots for assessing reporting bias. a) Recurrence rate. b) Operative times

Supplementary file 5.

## Data Availability

Direct contact to the corresponding author.
