# Correction to: Comprehensive meta‑analysis of surgical procedure for congenital diaphragmatic hernia: thoracoscopic versus open repair

**DOI:** 10.1007/s00383-024-05832-8

**Published:** 2024-08-31

**Authors:** Soichi Shibuya, Irene Paraboschi, Stefano Giuliani, Takafumi Tsukui, Andreea Matei, Maricarmen Olivos, Mikihiro Inoue, Simon A. Clarke, Atsuyuki Yamataka, Augusto Zani, Simon Eaton, Paolo De Coppi

**Affiliations:** 1 Stem Cell and Regenerative Medicine Section, Developmental Biology and Cancer Research & Teaching Department, Zayed Centre for Research Into Rare Disease in Children, Great Ormond Street Institute of Child Health, University College London, 30 Guilford Street, London, WC1N 1E UK; 2 Department of Pediatric General and Urogenital Surgery, Juntendo University School of Medicine, 3-1-3 Hongo, Bunkyo City, Tokyo, 113-8431 Japan; 3 Department of Biomedical and Clinical Science, University of Milano, Milan, Italy; 4 Department of Specialist Neonatal and Paediatric Surgery, Great Ormond Street Hospital for Children, NHS Foundation Trust, London, UK; 5 Division of General and Thoracic Surgery, The Hospital for Sick Children, Toronto, ON Canada; 6 Chelsea and Westminster NHS Foundation Trust, London, UK; 7 Department of Pediatric Surgery, Fujita Health University, Aichi, Japan; 8 Department of Surgery, University of Toronto, Toronto, ON Canada

**Correction to: Pediatric Surgery International (2024) 40:182** 10.1007/s00383-024-05760-7

In this article the wrong figure appeared as Fig. 1; the Fig. 1 should have appeared as shown below.

**Fig. 1:**
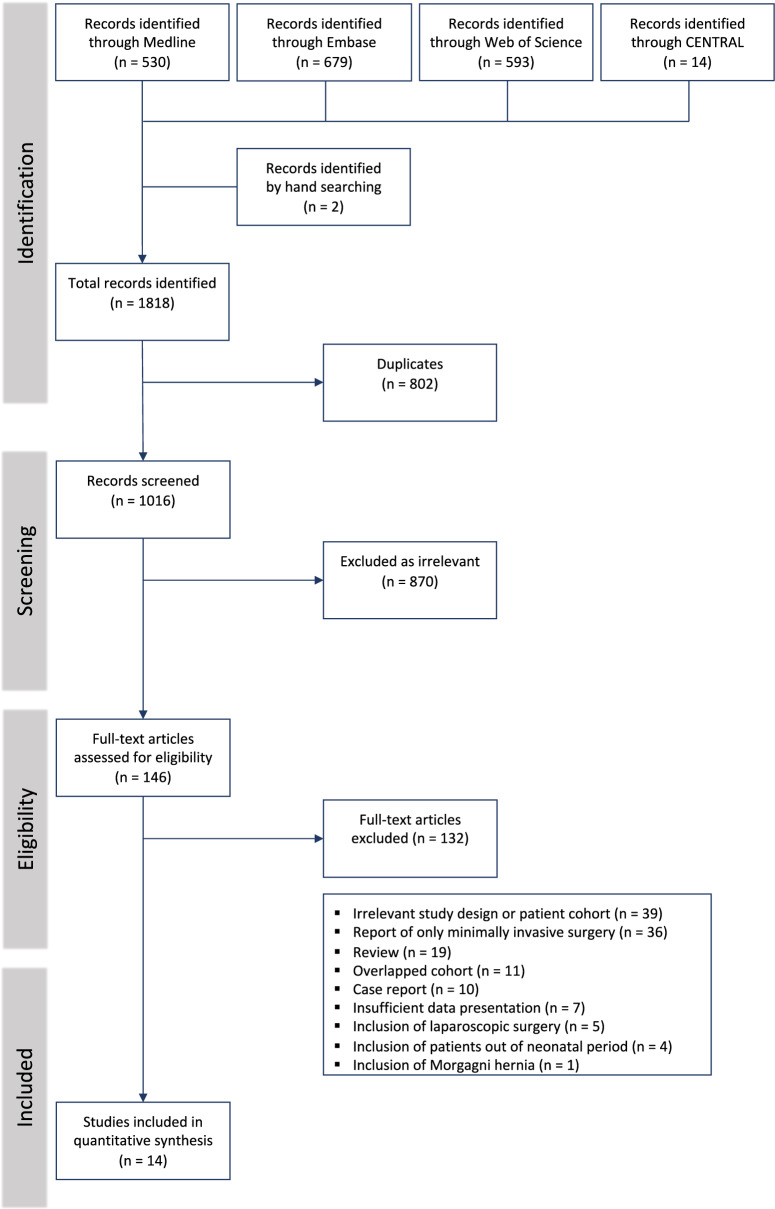
PRISMA flow chart

