# Supplementary material for: Comprehensive meta-analysis of surgical procedure for congenital diaphragmatic hernia: thoracoscopic versus open repair
Source: Pediatr Surg Int. 2024 Jul 9;40(1):182. doi: 10.1007/s00383-024-05760-7 (PMC11233350; doi:10.1007/s00383-024-05760-7)

Suppl.1 Comparisons of recurrence rate according to patch usage

a. Open patch repair vs primary closure for open surgery

Study or Subgroup	Patch repair Events	Patch repair Total	Primary closure Events	Primary closure Total	Weight	Odds Ratio M-H, Random, 95% CI	Year
McHoney et al. 2010	1	12	1	20	22.1%	1.73 [0.10, 30.45]	2010
Gander et al. 2011	0	16	0	3		Not estimable	2011
Nam et al. 2013	0	5	0	29		Not estimable	2013
Inoue et al. 2015	0	5	0	11		Not estimable	2015
Costerus et al. 2015	2	23	0	11	18.7%	2.67 [0.12, 60.54]	2015
Zani et al. 2017	7	40	0	67	21.8%	30.22 [1.68, 545.20]	2017
Criss et al. 2017	1	3	0	13	15.1%	16.20 [0.50, 522.43]	2017
Olivos et al. 2018	1	13	1	27	22.3%	2.17 [0.12, 37.64]	2018
Bawazir et al. 2021	0	2	0	28		Not estimable	2021
**Total (95% CI)**		**119**		**209**	**100.0%**	**5.15 [1.34, 19.86]**	
Total events	12		2				

Heterogeneity: $Tau^2 = 0.00$; $Chi^2 = 3.08$, df = 4 (P = 0.54); $I^2 = 0\%$
Test for overall effect: Z = 2.38 (P = 0.02)

Odds Ratio
M–H, Random, 95% CI

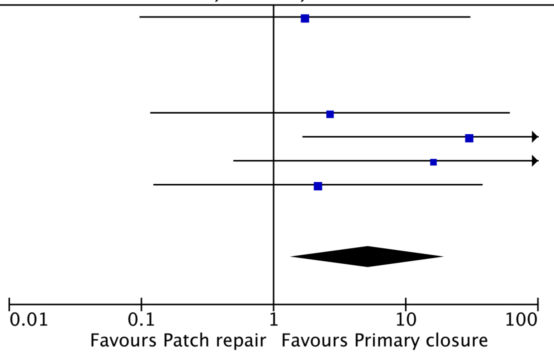

b. Thoracoscopic patch repair vs primary closure

Study or Subgroup	Patch repair Events	Patch repair Total	Primary closure Events	Primary closure Total	Weight	Odds Ratio M-H, Random, 95% CI	Year
McHoney et al. 2010	1	6	0	0		Not estimable	2010
Gander et al. 2011	3	12	3	14	17.8%	1.22 [0.20, 7.59]	2011
Nam et al. 2013	1	4	1	12	6.4%	3.67 [0.17, 77.55]	2013
Inoue et al. 2015	0	4	0	4		Not estimable	2015
Costerus et al. 2015	10	44	4	31	37.2%	1.99 [0.56, 7.03]	2015
Zani et al. 2017	2	5	5	21	14.1%	2.13 [0.27, 16.60]	2017
Criss et al. 2017	3	9	3	26	17.7%	3.83 [0.61, 24.02]	2017
Olivos et al. 2018	1	5	1	17	6.7%	4.00 [0.20, 78.79]	2018
Bawazir et al. 2021	0	0	1	11		Not estimable	2021
**Total (95% CI)**		**89**		**136**	**100.0%**	**2.25 [1.04, 4.87]**	
Total events	21		18				

Heterogeneity: $Tau^2 = 0.00$; $Chi^2 = 1.04$, df = 5 (P = 0.96); $I^2 = 0\%$
Test for overall effect: Z = 2.06 (P = 0.04)

Odds Ratio
M–H, Random, 95% CI

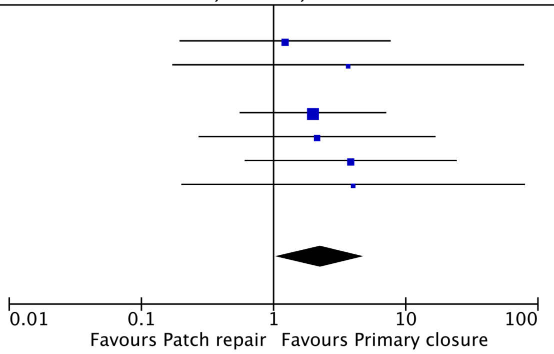

Supplement: Supplementary file 1 — Suppl.1 Comparisons of recurrence rate according to patch usage. a) Open patch repair vs primary closure in open surgery. b) Thoracoscopic patch repair vs primary closure in thoracoscopic surgery [file 383_2024_5760_MOESM1_ESM.pdf]
