# Supplementary material for: Comprehensive meta-analysis of surgical procedure for congenital diaphragmatic hernia: thoracoscopic versus open repair
Source: Pediatr Surg Int. 2024 Jul 9;40(1):182. doi: 10.1007/s00383-024-05760-7 (PMC11233350; doi:10.1007/s00383-024-05760-7)

Suppl.2 Subgroup analysis including only the cases later than 2011

a. Recurrence rate

	Thoracoscopy		Open			Odds Ratio	
**Study or Subgroup**	**Events**	**Total**	**Events**	**Total**	**Weight**	**M–H, Random, 95% CI**	**Year**
He et al. 2016	1	10	1	21	56.4%	2.22 [0.12, 39.64]	2016
Olivos et al. 2018	1	11	0	30	43.6%	8.71 [0.33, 230.79]	2018
Bawazir et al. 2021	0	14	0	14		Not estimable	2021
**Total (95% CI)**		**35**		**65**	**100.0%**	**4.03 [0.46, 35.10]**	
Total events	2		1				

Heterogeneity: $Tau^2 = 0.00$; $Chi^2 = 0.38$, df = 1 (P = 0.54); $I^2 = 0\%$
Test for overall effect: Z = 1.26 (P = 0.21)

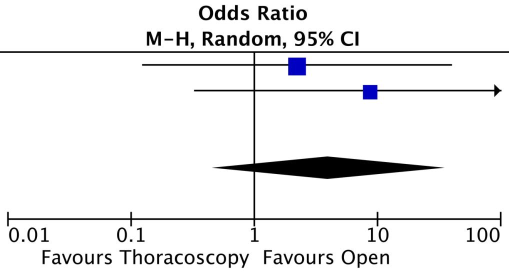

b. Operative times

	Thoracoscopy			Open				Mean Difference	
**Study or Subgroup**	**Mean**	**SD**	**Total**	**Mean**	**SD**	**Total**	**Weight**	**IV, Random, 95% CI**	**Year**
He et al. 2016	116	27	14	74	25	14	33.5%	42.00 [22.73, 61.27]	2016
Olivos et al. 2018	138.9	28.4	10	136.3	55.5	21	30.7%	2.60 [−26.95, 32.15]	2018
Bawazir et al. 2021	171.8	8.1	11	91.3	4.7	30	35.8%	80.50 [75.43, 85.57]	2021
**Total (95% CI)**			**35**			**65**	**100.0%**	**43.66 [0.66, 86.66]**	

Heterogeneity: $Tau^2 = 1339.31$; $Chi^2 = 38.67$, df = 2 (P < 0.00001); $I^2 = 95\%$
Test for overall effect: Z = 1.99 (P = 0.05)

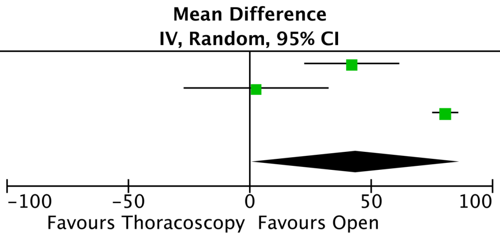

Supplement: Supplementary file 2 — Suppl.2 Subgroup analysis including only the cases later than 2011. a) Recurrence rate. b) Operative times (minutes) [file 383_2024_5760_MOESM2_ESM.pdf]
