# Supplementary material for: Comprehensive meta-analysis of surgical procedure for congenital diaphragmatic hernia: thoracoscopic versus open repair
Source: Pediatr Surg Int. 2024 Jul 9;40(1):182. doi: 10.1007/s00383-024-05760-7 (PMC11233350; doi:10.1007/s00383-024-05760-7)
Supplement: Supplementary file 3 — Suppl.3 Subgroup analysis including only the cases with a small defect. a) Recurrence rate. b) Operative times (minutes) *A small defect is defined as either of the following: 1) Type A or B defect according to The Congenital Diaphragmatic Hernia Study Group (Larry et al., 2013, J Pediatr Surg). 2) Maximum diameter of the defect is less than 5 cm [file 383_2024_5760_MOESM3_ESM.pdf]

Suppl.3 Subgroup analysis including only the cases with a *small defect

a. Recurrence rate

Study or Subgroup	Thoracoscopy Events	Thoracoscopy Total	Open Events	Open Total	Weight	Odds Ratio M-H, Random, 95% CI	Year
Inoue et al. 2015	0	8	0	16		Not estimable	2015
Criss et al. 2017	6	35	1	16	100.0%	3.10 [0.34, 28.20]	2017
**Total (95% CI)**		**43**		**32**	**100.0%**	**3.10 [0.34, 28.20]**	
Total events	6		1				

Heterogeneity: Not applicable
Test for overall effect: Z = 1.01 (P = 0.31)

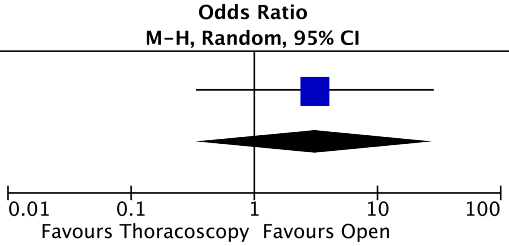

b. Operative times (minutes)

Study or Subgroup	Thoracoscopy Mean	Thoracoscopy SD	Thoracoscopy Total	Open Mean	Open SD	Open Total	Weight	Mean Difference IV, Random, 95% CI	Year
Inoue et al. 2015	212	35.8	8	115	26.1	16	50.1%	97.00 [69.09, 124.91]	2015
Criss et al. 2017	169.3	59.5	35	151	43	16	49.9%	18.30 [−10.55, 47.15]	2017
**Total (95% CI)**			**43**			**32**	**100.0%**	**57.74 [−19.39, 134.86]**	

Heterogeneity: $Tau^2 = 2887.10$; $Chi^2 = 14.76$, df = 1 (P = 0.0001); $I^2 = 93\%$
Test for overall effect: Z = 1.47 (P = 0.14)

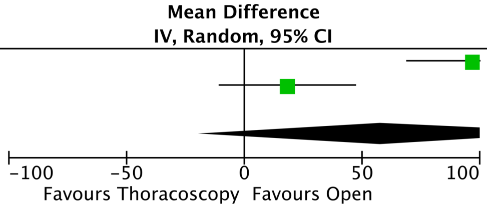

*A small defect is defined as either of the following:
1) Type A or B defect according to The Congenital Diaphragmatic Hernia Study Group (Larry et al., 2013, J Pediatr Surg).
2) Maximum diameter of the defect is less than 5 cm
