# Supplementary figures and images for: Comprehensive meta-analysis of surgical procedure for congenital diaphragmatic hernia: thoracoscopic versus open repair
Source: Pediatr Surg Int. 2024 Jul 9;40(1):182. doi: 10.1007/s00383-024-05760-7 (PMC11233350; doi:10.1007/s00383-024-05760-7)

Suppl.4 Funnel plots for assessing reporting bias

a. Recurrence rate

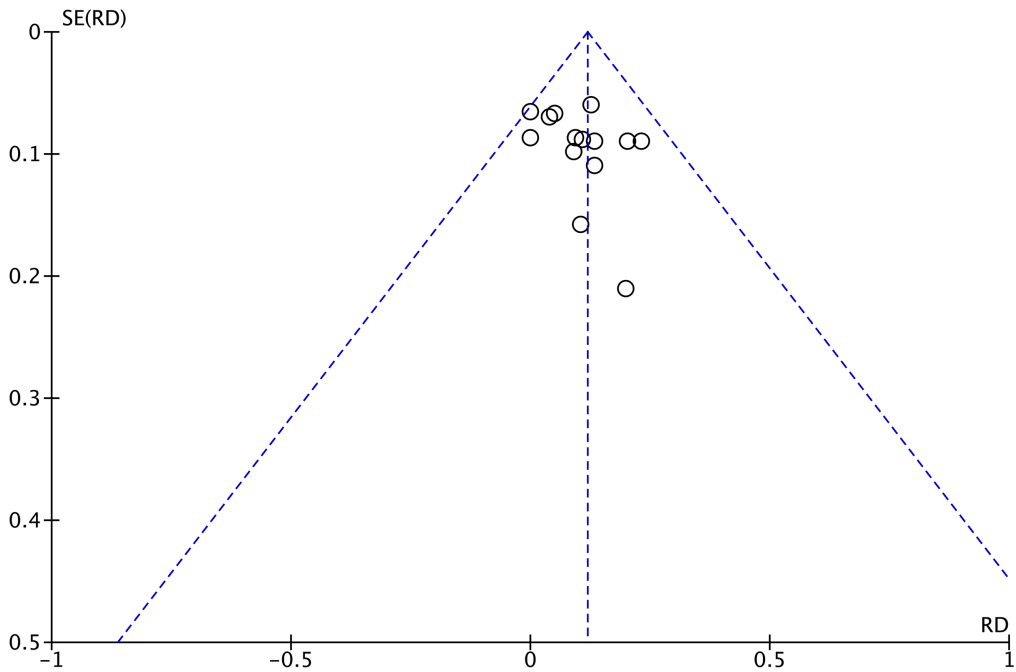

b. Operative times

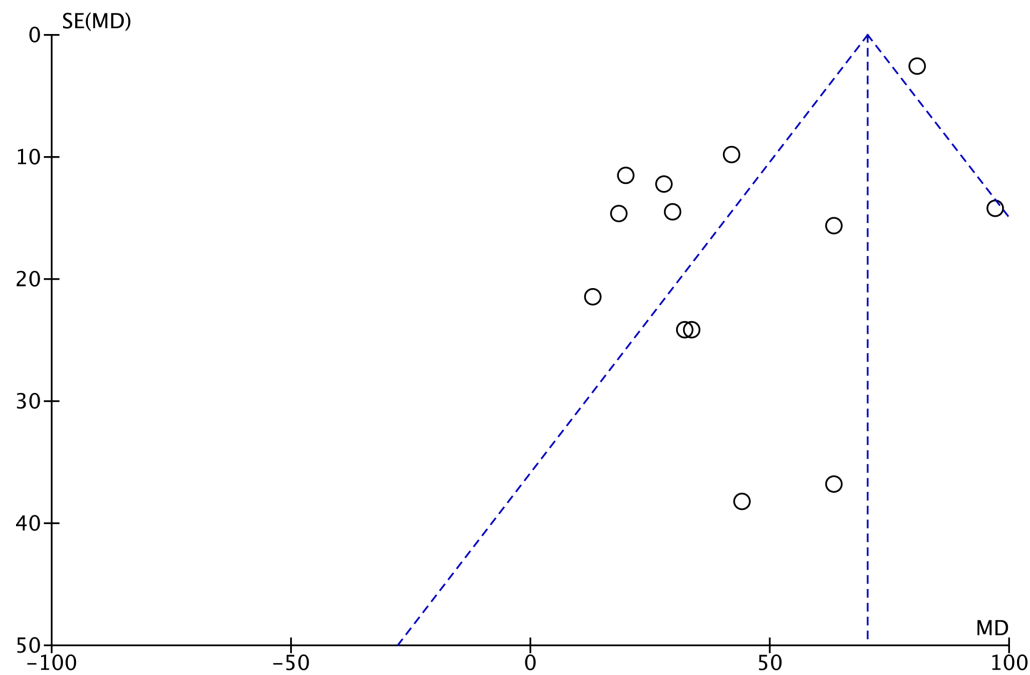

Supplement: Supplementary file 4 — Suppl.4 Funnel plots for assessing reporting bias. a) Recurrence rate. b) Operative times [file 383_2024_5760_MOESM4_ESM.pdf]
